# Simple geometry tribological study of osteochondral graft implantation in the knee

**DOI:** 10.1177/0954411917751560

**Published:** 2018-01-27

**Authors:** Philippa Bowland, Eileen Ingham, John Fisher, Louise M Jennings

**Affiliations:** Institute of Medical and Biological Engineering, School of Mechanical Engineering, University of Leeds, Leeds, UK

**Keywords:** Osteochondral graft, tribology, natural knee joint, friction simulator, friction, wear, cartilage, optical profiler

## Abstract

Robust preclinical test methods involving tribological simulations are required to investigate and understand the tribological function of osteochondral repair interventions in natural knee tissues. The aim of this study was to investigate the effects of osteochondral allograft implantation on the local tribology (friction, surface damage, wear and deformation) of the tissues in the natural knee joint using a simple geometry, reciprocating pin-on-plate friction simulator. In addition, the study aimed to assess the ability of osteochondral grafts to restore a low surface damage, deformation and wear articulation when compared to the native state. A method was developed to characterise and quantify surface damage wear and deformation of the opposing cartilage-bone pin surface using a non-contacting optical profiler (Alicona Infinite Focus). Porcine 12 mm diameter cartilage-bone pins were reciprocated against bovine cartilage-bone plates that had 6 mm diameter osteochondral allografts, cartilage defects or stainless steel pins (positive controls) inserted centrally. Increased levels of surface damage with changes in geometry were not associated with significant increases in the coefficient of dynamic friction. Significant damage to the opposing cartilage surface was observed in the positive control groups. Cartilage damage, deformation and wear (as measured by change in geometry) in the xenograft (2.4 mm^3^) and cartilage defect (0.99 mm^3^) groups were low and not significantly different (p > 0.05) compared to the negative control in either group. The study demonstrated the potential of osteochondral grafts to restore the congruent articular surface and biphasic tribology of the natural joint. An optical method has been developed to characterise cartilage wear, damage and deformation that can be applied to the tribological assessment of osteochondral grafts in a whole natural knee joint simulation model.

## Introduction

A wide variety of surgical approaches for the repair of early stage osteochondral defects are currently available.^1^ These range from treatments that aim to stimulate the growth of fibrous repair tissue (microfracture) to cell-based approaches such as autologous chondrocyte implantation, which aim to repair cartilage defects with a hyaline-like cartilage tissue. The transplantation of osteochondral autografts and allografts into the site of osteochondral defects has also been used as a surgical approach for the repair of cartilage and underlying bone defects in the knee since the early 1990s.^2^

The main aim of osteochondral graft surgery is to reconstruct the natural articulating surface and biphasic tribology of the joint, thereby restoring a low friction and wear articulation. In order for them to be successful, osteochondral grafts must possess adequate mechanical and tribological properties to withstand the complex loading environment within the natural knee, be biomechanically stable and become integrated into the natural tissues over time. Furthermore, the tribological properties of the graft, subsequent repair tissues and the resultant level of joint congruency achieved should not compromise the integrity of the surrounding and opposing cartilage surfaces. Osteochondral grafting aims to repair the underlying supporting bone structure and restore a near frictionless articulating surface; therefore, the tribological performance of osteochondral grafts in the natural joint is a key factor in determining their success in the short and long term.

The clinical application and outcome of osteochondral grafting is limited by a number of factors including defect size and tissue availability, donor site morbidity, recipient site congruity, graft integration and fibrocartilage in-growth.^3–5^ Tissue engineering of osteochondral scaffolds and constructs has the potential to overcome the current limitations of osteochondral graft (autologous and allogeneic) transplantation and provide a novel, early intervention repair therapy with improved long-term outcomes.

Simple geometry, pin-on-plate tribological test methods have commonly been used to study the tribology of cartilage;^6–10^ similarly, the use of these test methods has also been extended to evaluate the tribology of potential cartilage biomaterials^11–15^ and engineered cartilage substitutes.^16–20^ Small-scale, in-vitro pin-on-plate test methods (while not replicating the geometry or complex motions of the natural knee) allow for the direct control of experimental variables such as normal load, sliding distance and velocity, contact pressure and tissue unloading intervals that ultimately dictate the outputs under investigation.^10^ Despite the long-standing clinical application of osteochondral grafts, there has been limited preclinical evaluation of their tribological performance in the natural knee to date, in either simple geometry or whole joint models.^21,22^

The overall aim of this study was to investigate the effects of osteochondral graft implantation on the local tribology (friction, surface damage, wear and deformation) of the natural knee joint using a simple geometry, reciprocating pin-on-plate animal tissue model. The objectives of this study were to (a) assess the ability of osteochondral grafts to restore low levels of cartilage surface damage, wear and deformation (on the opposing surface) when compared to the native state and (b) develop a method to quantify changes in surface geometry, as a measure of cartilage surface damage and wear using an optical profiler (Alicona Infinite Focus).

The study represents the first published study investigating the tribology of xenografts (natural cartilage-bone grafts) in a simple geometry tribological tissue model of the knee. In order to develop novel tissue engineered scaffolds and constructs (such as regenerative early intervention therapies in the knee), there is the requirement to understand their mechanical and tribological function in the natural knee. Additionally, it is important to determine how the complex range of variables in the knee joint as a biomechanical system interacts with the design of the intervention to determine the resultant tribology. Robust preclinical test methods involving tribological simulations are therefore required to investigate and understand the tribological function of osteochondral repair interventions in the tissues of the natural knee. The study was conducted as a preliminary, simple geometry tribological investigation to understand the effects of osteochondral graft implantation on the tribology of the articulating surfaces in the knee and to inform the development of a whole natural knee joint simulation model.

## Materials

Osteochondral plates were harvested from the patella-femoral groove of skeletally mature (18 month old) bovine femurs; bovine femurs were used for the harvest of osteochondral plates since porcine femoral grooves were too small to harvest plates of the required dimensions for testing. A hand saw and purposely designed jig were used to harvest flat rectangular plates with dimensions of 45 × 17 × 7 mm. Reciprocating cylindrical osteochondral pins, 12 mm in diameter, were harvested from the weight bearing region of porcine (4–6 month old) medial femoral condyles using a drill aided corer. The harvest location of the reciprocating pins resulted in a slightly curved pin surface, representing a similar geometry to the whole femoral condyle in the natural knee joint. Flat ended porcine osteochondral xenografts, 6 mm in diameter, were harvested from the patella-femoral groove of porcine femurs using a drill aided corer. The osteochondral graft positive controls used in the tests were 6 mm diameter, cylindrical stainless steel (type 316) pins (surface roughness (Ra) of 0.005 ± 0.001 mm). In order to insert the 6 mm diameter porcine xenografts or stainless steel control pins into the bovine osteochondral plates, a 6 mm recipient hole had to be drilled into the central region of the plate. The osteochondral plate was then placed onto the base plate of the fixture such that a grub screw on the base plate and the recipient hole in the osteochondral plate were aligned. The 6 mm graft (stainless steel or porcine xenograft) was then push-fit into the recipient hole; the height of the grub screw was then altered from the reverse side of the base plate until the graft sat either flush or 1 mm proud of the cartilage surface. To create cartilage defects in the plates, a 6 mm diameter biopsy punch was used to insert a 6 mm cartilage defect into the centre of the osteochondral plates. A biopsy punch was inserted into the cartilage down to the subchondral bone and used to core out a circular disc of cartilage. The cartilage disc was then ejected from the biopsy punch by depressing the release mechanism on the end of the punch. Tissue samples were kept hydrated throughout the harvesting procedures using phosphate buffered saline (PBS; MP Biomedicals LLC, Santa Ana, USA) and stored until required for testing on PBS soaked tissue paper at −20 °C. PBS was prepared as per the manufacturers guidelines by dissolving one tablet per 100 mL of sterile water. Samples were removed from storage prior to testing and thawed at room temperature.

## Experimental design

**Figure 1.**
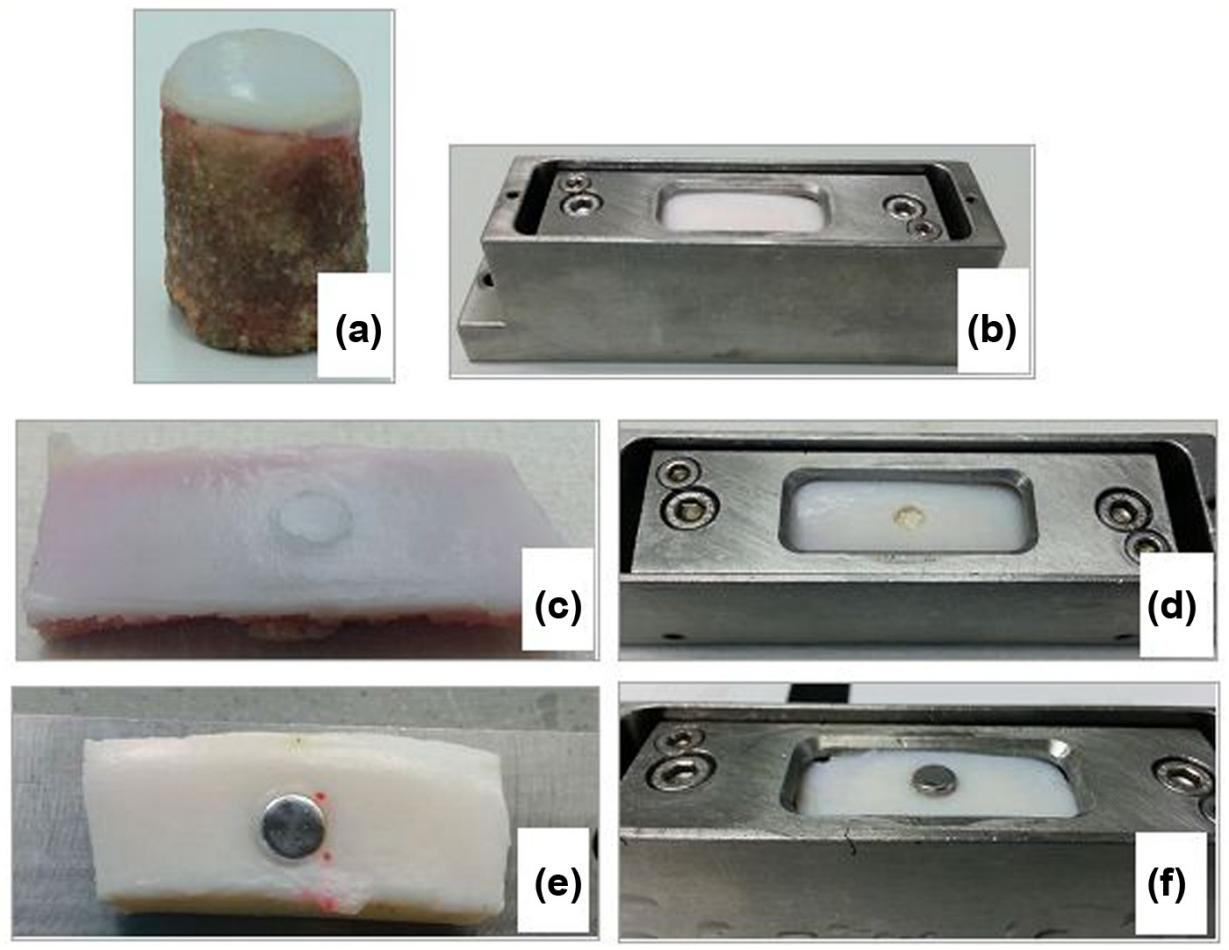
Images of the 12 mm diameter reciprocating pin (a): an osteochondral plate with no graft/defects inserted (negative control group) within the lubricant bath (b); xenograft group (c); cartilage defect group (d); Positive control group 1 (stainless steel pins inserted flush) (e); Positive control group 2 (stainless steel pins inserted 1 mm proud) (f).

The experimental setup for all test groups consisted of a curved ended, 12 mm diameter porcine osteochondral pin, reciprocating against a flat bovine osteochondral plate (Figure 1).

Each osteochondral plate and reciprocating pin pair were initially run under the test conditions described later as a negative control for a duration of 3 h (Figure 1). Following this, the specimens were allowed to recover unloaded for 1 h before either a 6 mm diameter xenograft, a cartilage defect or a stainless steel pin was inserted in the centre of the osteochondral plate (Figure 1). The negative controls consisted of an osteochondral plate with no pins, defects or interventions inserted (native state; n = 24). Test samples were then run as either a positive control or one of the experimental groups for a further 3 h. Positive control groups consisted of stainless steel pins inserted flush with the articular surface of the osteochondral plate (Positive control group 1; n = 6) and 1 mm proud of the articular cartilage surface (Positive control group 2; n = 6) (Figure 1). A further two experimental groups were tested: Experimental group 1 – cartilage defects inserted down to subchondral bone (n = 6); Experimental group 2 – porcine xenografts inserted flush with the articular cartilage surface (n = 6). All experimental and control group tests were performed in a lubricant of PBS + 25% (v/v) newborn calf serum (Gibco Life Technologies, Paisley, UK) for a duration of 3 h.

## Methods

### Simple geometry tribological tests

A reciprocating motion, pin-on-plate friction rig was used for all tribological tests; a full description of the friction rig is provided by Northwood et al.^23^ Reciprocating osteochondral pins (12 mm diameter) were fixed in a static loaded pin holder with the cartilage surface contacting the cartilage surface of the osteochondral plate. Osteochondral plates were secured in a lubricant bath that was fixed to the reciprocating platen of the friction rig. The frictional force between the pin and plate samples was transmitted to a piezoelectric sensor; the corresponding output voltage of the piezoelectric force sensor was relayed to a digital charge amplifier and stored on a PC using a data acquisition unit. Friction tests were performed using a sliding velocity of 10 mm s^−1^, a stroke length of 20 mm and applied load of 120 N; all tests were performed in a lubricant of PBS + 25% (v/v) newborn calf serum (Gibco Life Technologies, Paisley, UK) for a duration of 3 h.

The output voltage of the piezoelectric sensor was used to calculate the dynamic friction coefficient (μ) from the middle of the stroke using a known calibration factor; the piezoelectric sensor was calibrated by applying a range of known forces (loads) to the pin holder in the direction of motion of the reciprocating platen. A calibration curve was produced by plotting the average voltage (V) readings against the cumulative load (N) applied and a linear trend line fitted to the data. The gradient and y-intercept of the trend line were used to derive the dynamic friction coefficient (μ) using equation (1)

(1) $$\begin{array}{c} \mathrm{Dynamic Friction Coefficient}\left(\mu \right)=\frac{\mathrm{Friction Force}\left(N\right)}{\mathrm{Applied Load}\left(N\right)}\\ =\frac{\left(\frac{\left(\frac{\mathrm{Average Maximum Voltage}-\mathrm{Average Minimum Voltage}}{2}\right)-\mathrm{Y Intercept}}{\mathrm{Gradient of Calibration}}\right)}{\mathrm{Load}} \end{array}$$

**Figure 2.**
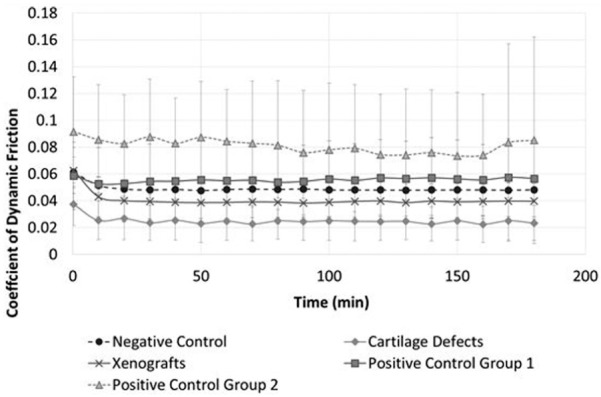
Mean coefficient of dynamic friction plotted at 10 min time intervals (mean ± 95% confidence limits; n = 6 per group).

The dynamic friction coefficient was recorded at 60 s intervals; for clarity, the data in Figure 2 are plotted at 10 min intervals as the mean ± 95% confidence limits. The means of each paired negative control and the associated experimental or positive control group were compared using a paired students t-test at time intervals of 60, 120 and 180 min to determine any significant difference (p = 0.05) in coefficient of dynamic friction.

The relative change in coefficient of dynamic friction between each experimental group (F_E_) and paired negative control (F_C_) was compared by calculating the change in friction (ΔF) using equation (2)

(2) $$\Delta F=F_{E}-F_{C}$$

### Assessment and quantification of articular cartilage surface damage, wear and deformation

The surface geometry of the opposing articular cartilage surface (reciprocating osteochondral pin) to the insertion site of the grafts, defects or stainless steel pins was assessed and quantified using an Alicona Infinite Focus G5 optical 3D micro coordinate and surface roughness measurement device. Following each experimental and control group test, the surfaces of the reciprocating pins were replicated using Microset 101 RF (Microset Products Ltd, UK) high-resolution silicone replicating compound. The opposing cartilage surface replicas were scanned on the Alicona Infinite Focus using a x10 objective, 358 nm vertical resolution and 7.54 µm lateral resolution (contrast and exposure settings were optimised as appropriate per sample) to produce 3D image data sets (3D reconstructions of the original sample surface).

Surface damage, wear and deformation were quantified using the Alicona Infinite Focus by measuring changes in the geometry of the cartilage surface (change in volume extending beneath the cartilage surface). The change in volume (mm^3^) extending below the articular cartilage surface was used as a measure of cartilage surface damage, wear and deformation occurring during the pin-on-plate tests (it was not possible to assume that changes in surface geometry were solely due to wear arising from removal of material/tissue; changes in surface geometry may also have been attributable to tissue deformation or damage without the loss of material). The volume (mm^3^; below cartilage surface level) and depth of damage, wear and deformation were measured from the 3D image data sets, using the analysis software IF Measure Suite Version 5.1 (Alicona, Austria). All volume calculations were performed using a “Top Cover” filter within the analysis software.

A group of six negative control specimens were analysed using the Alicona Infinite Focus to determine any subsequent changes in the surface geometry during the negative control tests. The volume extending below the sample surface was quantified for a sample area of 30 mm^3^ (25% reciprocating pin surface area) for each pin and compared between the native state (untested specimen) and following the negative control test.

The mean volume (mm^3^) extending below the surface of the negative control samples (0.1 mm^3^) was used as a baseline for statistical comparison (independent samples t-test; p = 0.05) with the experimental and positive control groups to determine any significant changes in the cartilage surface geometry. One-way analysis of variance (ANOVA) was used to compare the mean depth of surface damage, wear and deformation observed between the positive control, xenograft and cartilage defects groups at the p = 0.05 significance level. The data set associated with this article is openly available from the University of Leeds Data Repository.^24^

## Results

### Dynamic friction

The negative control samples maintained a low constant coefficient of dynamic friction (0.049 ± 0.007) throughout the test duration following a small initial decrease during the first 10 min (Figure 2). No significant differences (p > 0.05; paired t-test) in dynamic friction were recorded between the negative controls and any of their paired experimental or positive control group tests at any time point analysed (60, 120 and 180 min).

**Figure 3.**
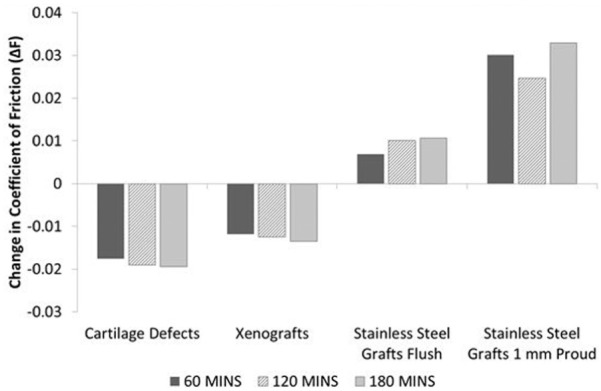
Mean change in coefficient of dynamic friction between the negative control tests and each paired experimental or positive control group test at 60, 120 and 180 min (n = 6 per group). Change in dynamic friction (ΔF) calculated as experimental friction (F_E_) minus negative control friction (F_C_).

The mean change in the coefficient of dynamic friction between the negative control groups and their paired experimental or positive control group is presented in Figure 3. A positive increase in dynamic friction was recorded for Positive control group 1 (stainless steel pins inserted flush) and Positive control group 2 (stainless steel pins inserted 1 mm proud) at time points 60, 120 and 180 min when compared to the paired negative control tests. Positive control group 2 exhibited the greatest overall change in recorded friction across all groups. The mean coefficient of friction for the cartilage defect and xenograft groups was lower than the negative controls; the largest decrease in coefficient of dynamic friction was recorded in the cartilage defect group at 180 min (–0.019).

### Articular cartilage surface damage, wear and deformation

Visual inspection of the negative control 3D images indicated that there were no apparent changes in the cartilage surface geometry when compared to the untested samples. No significant difference (p = 0.538, paired t-test) was present in the volume extending below the cartilage surface (a measure of damage, wear and deformation) between the untested (0.15 mm^3^) and negative control test samples (0.098 mm^3^), indicating cartilage surface damage and wear were not present.

**Figure 4.**
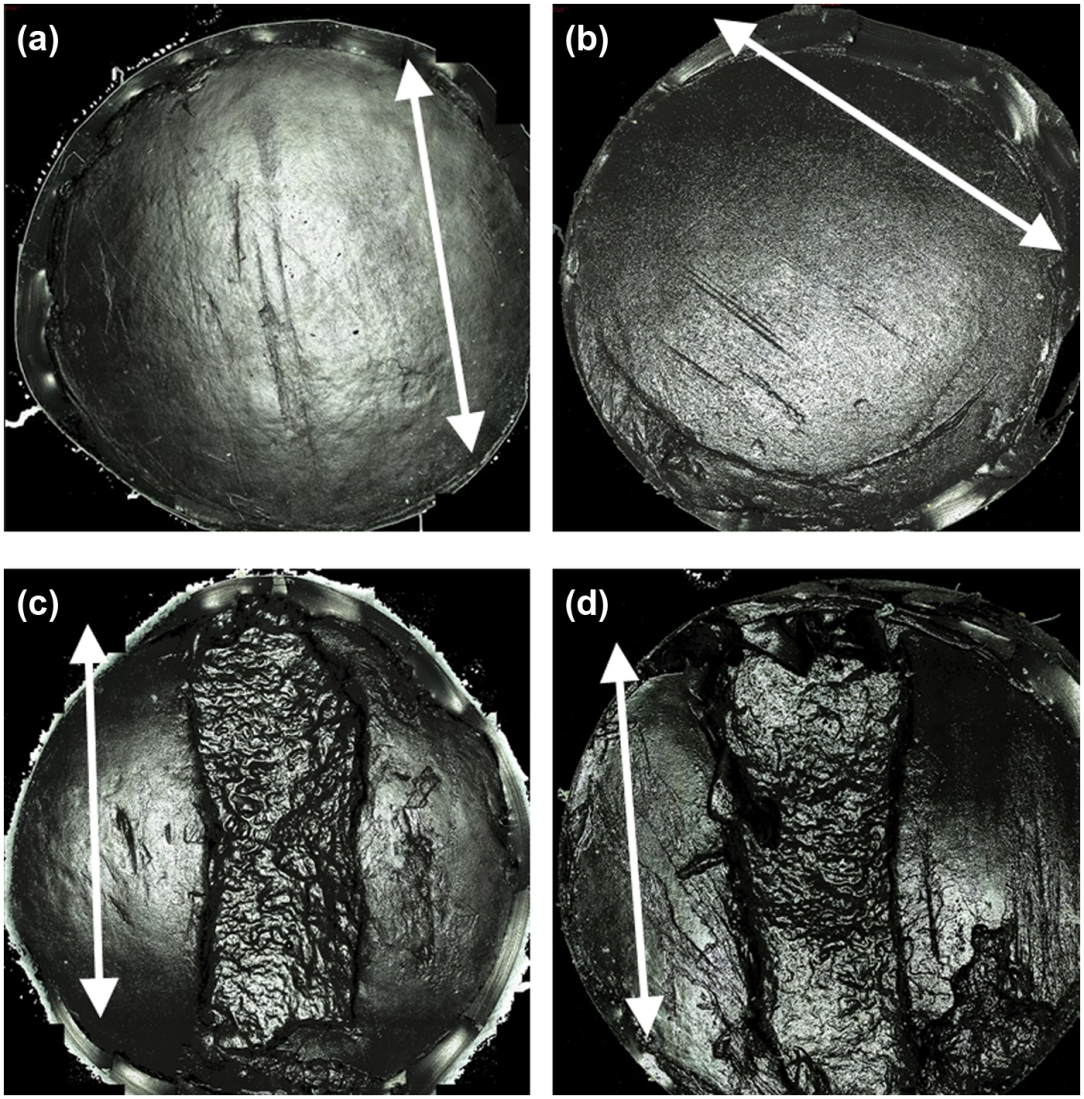
Example Alicona Infinite Focus scan images of the reciprocating 12 mm diameter pin surface with arrows indicating sliding direction: (a) cartilage defect group, (b) xenograft group, (c) Positive control group 1 (stainless steel pins flush) and (d) positive control group 2 (stainless steel pins 1 mm proud).

**Figure 5.**
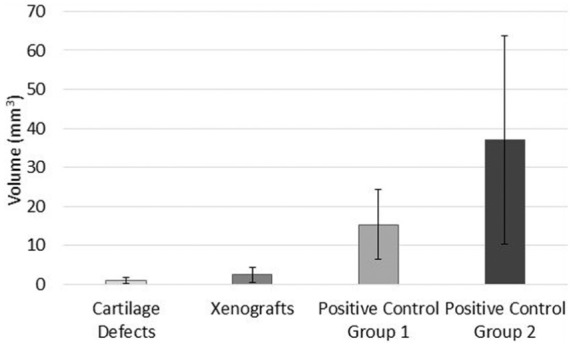
Mean volume (mm^3^; volume extending beneath cartilage surface) of articular cartilage surface damage, wear and deformation (mean ± 95% confidence intervals; n = 6 per group) measured using the Alicona Infinite Focus.

Positive control groups 1 and 2 sustained severe cartilage damage, wear and deformation, as shown in Figure 4. Damage, wear and deformation in the positive control groups generally consisted of large, steep flanked cartilage lesions stretching across the diameter of the pin surface, accompanied by multiple deep scratches on the surrounding cartilage surface. The mean volume of cartilage damage and wear in Positive control groups 1 and 2 was significantly greater (p = 0.021 and p = 0.042; independent t-test) than the negative controls at 15.3 and 37.1 mm^3^, respectively (Figure 5).

The cartilage defect group had the smallest mean volume of damage, wear and deformation at 0.99 mm^3^; similarly, the volume of the xenograft group was also low at 2.4 mm^3^. No significant difference (p > 0.05) was present between the negative control group (0.098 mm^3^) and both the cartilage defect (p = 0.162; independent t-test) and xenograft (p = 0.188; independent t-test) groups. Damage, wear and deformation in the cartilage defect and xenograft groups consisted of visible areas of increased surface roughness, scratches in the central region of the pin surface orientated parallel with the direction of translation and/or small, shallow cartilage lesions with irregular boundaries (Figure 4).

**Figure 6.**
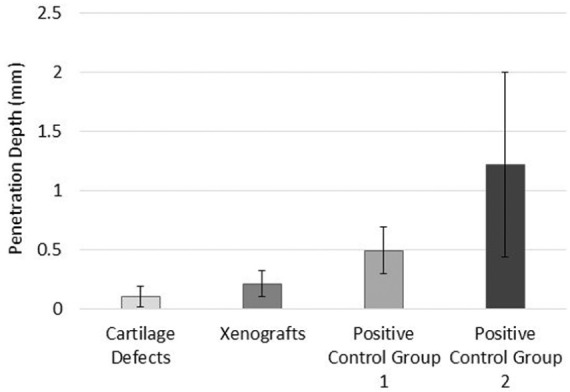
Mean penetration depth of articular cartilage surface damage, wear and deformation (mean ± 95% confidence intervals; n = 6 per group) measured using the Alicona Infinite Focus.

The cartilage defect and xenograft groups had the smallest penetration depths of damage and wear at mean depths of 0.11 and 0.21 mm, respectively (Figure 6); the group means were not significantly different (p = 0.986; ANOVA). Positive control group 2 had the largest mean depth of damage, wear and deformation at 1.22 mm and this was significantly greater (p < 0.05; ANOVA) than the cartilage defect and xenograft groups (Figure 6). Positive control group 1 also had a greater mean depth (0.49 mm) of damage, wear and deformation than the cartilage defect and xenograft groups; however, these differences were not significant (p > 0.05; ANOVA).

## Discussion

This study is the first to investigate the effects of cartilage defects and the implantation of osteochondral grafts on the local tribology. The constant low coefficient of dynamic friction (0.049 ± 0.007) demonstrated by the negative control group was similar to values reported in previous studies.^7,13,14,25,26^ The constant low coefficient of friction and the absence of surface damage and wear in the negative control group highlight the biphasic behaviour of cartilage and its intrinsic ability to maintain interstitial fluid load support and therefore, a low friction and wear articulation. The reciprocating motion of the articular cartilage plate promoted the maintenance of biphasic fluid load support by allowing for interstitial fluid rehydration during the unloaded phase of the cycle.^8^

Overall, there were no significant differences in dynamic friction (p > 0.05, paired t-test) between the negative controls and the paired experimental or positive control group tests (Figure 2); furthermore, increased levels of articular cartilage surface damage and wear were not associated with significant increases in the coefficient of dynamic friction. The positive control groups exhibited the highest levels of friction, with a small increase in the coefficient of friction observed between 60 and 180 min (Figure 3). The cartilage defect and xenograft groups demonstrated a lower mean coefficient of friction in comparison to the negative controls, with the greatest decrease in frictional coefficient (–0.019) recorded in the cartilage defect group at 180 min. The lower levels of friction recorded in the cartilage defect group were likely attributable to a reduction of contact area and increased fluid volume in the articulation, resulting in increased fluid load support of the applied load. These factors were also likely to have contributed to the low level of cartilage damage, wear and deformation (0.99 mm^3^) measured in the cartilage defect group (Figure 5).

The xenograft group displayed a low level of overall damage, wear and deformation at 2.4 mm^3^; general patterns in the damage and wear observed, included areas of surface roughness, scratching or small shallow cartilage lesions (mean depth 0.2 mm); similar patterns were also noted for the cartilage defect group (Figure 4). There were no significant differences (p > 0.05) in the volume of damage and wear (volume extending below the cartilage surface) when compared to the negative control (0.1 mm^3^). Overall, the mean volume of cartilage damage and wear measured for the xenograft group was low, indicating that following implantation, osteochondral grafts have the potential to restore a congruent articular surface and some degree of biphasic lubrication, resulting in a low friction articulation with low levels of resultant damage, wear and deformation. The insertion of osteochondral grafts into the natural contour of the osteochondral plates introduces a discontinuous articulating surface at the circumference of the grafts and defects. The translation of the reciprocating pin over the boundary region between the plates and grafts was believed to have been responsible for the damage, wear and deformation sustained in the xenograft group due to the presence of edge effects.

The majority of samples (n = 4) within the xenograft group had low levels of damage, wear and deformation at a mean volume of 0.13 mm^3^, predominately consisting of areas of noticeable surface roughness and scratching. In contrast, two samples within the group had a mean volume of 5.8 mm^3^, consisting of small isolated cartilage lesions. The difference in the volume and pattern of damage, wear and deformation was thought to be attributable to misalignment of the osteochondral grafts, resulting in protruding grafts that were subject to disproportionate loading in comparison with other samples in the group. The insertion of stainless steel pins 1 mm proud (Positive control group 2) resulted in cartilage damage, wear and deformation of greater mean volume and depth when compared with stainless steel grafts inserted flush (Positive control group 1). The large confidence interval range (Figures 5 and 6) within Positive control group 2 was attributable to one sample with a wear volume of 93 mm^3^. The surface area of the lesion was comparable to the group average; however, the depth was significantly greater (2.7 mm). The larger volume of cartilage damage, wear and deformation recorded for this sample was likely attributable to misalignment of the stainless steel pin, resulting in a protrusion greater than 1 mm above the cartilage surface.

The simple geometry pin-on-plate model used in this study was confined to unidirectional sliding motion and constant loading of the reciprocating pin. Combined rolling and sliding motions of the femoral condyles in the natural knee result in dynamic loading on both articular surfaces within the tibiofemoral joint; the reciprocating pin in contrast to the whole joint is a small discontinuous surface that is constantly loaded. The experimental geometry used within this study is thought to increase the rate of fluid loss away from the contact zone and reduce intrinsic fluid load support when compared to the natural tibiofemoral joint. The continuous loading of the reciprocating pin (opposing cartilage surface to graft/defect site) and the associated reduction in fluid load support essentially creates a worst case scenario when investigating the effects on friction, damage and wear associated with the presence of osteochondral grafts and cartilage defects.

The study presented a method for the assessment and characterisation of articular cartilage damage and wear using focus variation technology (Alicona Infinite Focus). The Alicona Infinite focus facilitated detailed 3D visual characterisation and quantitative assessment of changes in cartilage surface topography using one integrated system. The evaluation of cartilage damage, wear and deformation in previous simple geometry tribological studies has been mainly limited to the use of contacting stylus and laser surface profilometry, assessing changes in parameters such as surface roughness (Ra).^9,13,14,26–28^ The nature of surface profilometry methods such as contacting surface profilometry requires that additional techniques such as scanning electron microscopy and micro-MRI be utilised in order to image and visually assess cartilage wear, damage and deformation.^9,27,28^

Future work will focus on the development of a preclinical whole joint knee simulation model for the tribological assessment of osteochondral grafts and scaffolds as potential early interventions for the repair of osteochondral defects; cartilage wear, damage and deformation will be characterised and quantified using the optical method developed within this study.

## Conclusion

The study demonstrated the potential for osteochondral grafts to restore the congruent articular surface and biphasic tribology of the natural joint without introducing significant changes in friction, damage and wear when compared to the native state. The results and knowledge gained from simple geometry tribological tests can be used to inform the development of robust and stratified whole joint simulation models; furthermore, the results may act as a useful baseline to which the outcomes of whole joint simulation tests can be compared.
